# Classification of maize seed hyperspectral images based on variable-depth convolutional kernels

**DOI:** 10.3389/fpls.2025.1599231

**Published:** 2025-06-06

**Authors:** Yating Hu, Hongchen Zhang, Changming Li, Qianfu Su, Wei Wang

**Affiliations:** ^1^ College of Information Technology, Jilin Agricultural University, Changchun, China; ^2^ Engineering Technology R & D Center, Changchun Guanghua University, Changchun, China; ^3^ Institute of Plant Protection, Jilin Academy of Agricultural Sciences (Northeast Agricultural Research Center of China), Changchun, China

**Keywords:** variable-depth convolutional kernels, 3D convolutional kernel, CNN, corn, hyperspectral image, variety identification

## Abstract

**Introduction:**

Accurate classification of corn seeds is vital for the effective utilization of germplasm resources and the improvement of seed selection and breeding efficiency. Traditional manual classification methods are labor-intensive and prone to errors. In contrast, machine learning techniques—particularly convolutional neural networks (CNNs)—have demonstrated superior performance in terms of classification accuracy, robustness, and generalization. However, conventional hyperspectral data processing approaches often fail to simultaneously capture both spectral and textural features effectively.

**Methods:**

To overcome this limitation, we propose a novel convolutional neural network architecture with a variable-depth convolutional kernel structure (VD-CNN). This design enables the network to adaptively extract continuous spectral features by modulating kernel depth, while simultaneously capturing fine-grained textural patterns through hierarchical convolutional operations. In our experiments, we selected eight widely cultivated corn seed varieties and collected hyperspectral images for 100 seeds per variety. A four-layer CNN framework was constructed, and a total of 12 models were developed by varying the convolutional kernel depth to evaluate the impact on classification performance.

**Results:**

Experimental results show that the proposed VD-CNN achieves optimal performance when the convolutional kernel depth is set to 15, attaining a training accuracy of 98.65% and a test accuracy of 96.97%. To assess the generalization ability of the model, additional experiments were conducted on a publicly available rice seed hyperspectral dataset. The VD-CNN consistently outperformed existing benchmark models, improving the classification accuracy by 3.14% over the best baseline. These results validate the robustness and adaptability of the proposed architecture across different crop species and imaging conditions.

**Discussion:**

These findings demonstrate that the proposed VD-CNN effectively captures both spectral and textural features in hyperspectral data, significantly enhancing classification performance. The method offers a promising framework for hyperspectral image analysis in seed classification and other agricultural applications.

## 1 Introduction

Corn is one of the three major cereal crops worldwide and serves as a vital feed source and industrial raw material (Li et al., 2025). The quality of corn seeds directly impacts yield, food security, and the agricultural economy. However, with the rapid advancement of hybrid corn seed production technology, challenges related to seed purity have become increasingly prominent. The complexity of hybrid breeding systems, large-scale commercial seed multiplication, and potential for unintended cross-pollination have collectively contributed to a noticeable decline in seed purity in practical agricultural applications. This decline not only increases the risk of varietal admixture and identity confusion but also undermines the consistency of crop performance and ultimately leads to reduced yield and economic loss for producers. Therefore, accurate identification of corn seed varieties serves as the foundation for effective purity assessment and is vital for supporting downstream measures aimed at preserving genetic integrity and ensuring stable crop production (Xue et al., 2025).

Traditional identification methods, such as morphological analysis, molecular biology techniques, and genetic markers, are labor-intensive, time-consuming, and require specialized expertise (Yu et al., 2018). Moreover, these approaches often cause irreversible damage to samples, making them unsuitable for rapid and non-destructive quality assessment in industrial settings (Yang et al., 2018). Consequently, researchers have focused on developing fast, non-invasive techniques for seed classification and identification (Zhu et al., 2025).

In recent years, researchers have extensively explored non-destructive detection techniques that leverage the morphological and optical properties of seeds. These methods include X-ray diffraction (Ahmed et al., 2018), laser speckle analysis (Sutton and Punja, 2017), near-infrared spectroscopy (Li et al., 2018), multispectral imaging (MSI) (Cheng et al., 2024), hyperspectral imaging (HSI) (Geng et al., 2013), and Raman spectroscopy (Seo et al., 2016). Among these, both MSI and HSI have gained significant attention for their ability to combine imaging and spectral analysis. MSI captures reflectance information at a limited number of discrete wavelengths, offering a balance between data volume and analytical performance, which makes it suitable for rapid and cost-effective applications. HSI, on the other hand, is a more advanced technique that provides continuous spectral data across a broad range of wavelengths, enabling the simultaneous acquisition of detailed spectral and spatial information from the target subject (He et al., 2019). This high-resolution capability makes HSI particularly powerful for identifying subtle differences in seed composition and quality. In recent years, HSI technology has achieved significant research advancements in the field of remote sensing. To improve semantic segmentation performance across large-scale geospatial datasets, Li et al. (Pang et al., 2025) proposed SegEarth, which enhances pixel-level reasoning and benefits HSI tasks through transferable pretraining. Addressing the challenge of scarce annotations in HSI classification, Pang et al. (Sun et al., 2025) also introduced SPECIAL, which applies CLIP to align HSI spectral features with textual descriptions, enabling zero-shot classification of unseen categories. Furthermore, for temporal HSI analysis, Sun et al. (Li et al., 2025) developed the Mask Approximation Net (MAN), a diffusion-based model that generates detailed natural language captions to describe changes captured in multitemporal HSI data. In addition to the field of remote sensing, hyperspectral imaging (HSI) has also yielded numerous application results in desktop-level applications, such as monitoring the moisture content of tea leaves, assessing citrus fruit maturity, and detecting pesticide residues in vegetables (Wang and Song, 2023). Furthermore, HSI has been extensively employed in non-destructive testing of crop seed varieties, quality assessment, and viability evaluation (Bo et al., 2022). Manggala et al. (2023) provided a comprehensive review of pesticide residue detection techniques and emphasized the potential of HSI, especially when integrated with machine learning, to accurately identify and map residues across crop surfaces. Despite its promise, the complexity and cost of HSI limit its adoption among smallholder farmers. To address similar challenges in plant health monitoring, Putra et al. (2022)) proposed a low-cost, smartphone-based optical sensing system combined with deep neural networks to estimate *in-situ* chlorophyll content in maize, achieving results comparable to SPAD meters.

In recent years, the combination of traditional machine learning and spectroscopy has been widely applied to corn seed identification (Saha and Manickavasagan, 2021). Zhang et al. (Zhao et al., 2018) proposed an incremental learning model using various classifiers to identify five corn seed varieties based on hyperspectral images, achieving an accuracy close to 100%. Fu et al. (Zhang et al., 2022) employed HSI with an SSAE-CS-SVM model to distinguish four corn varieties, obtaining a test accuracy of 95.81%. Wang et al. (Fu et al., 2022) developed a fusion model integrating dual-band ratio images and texture features to identify seeds harvested over four different years, achieving an accuracy of 97.5%. Other studies by Tu et al. (Wang et al., 2022) and Hu et al. (Tu et al., 2022) further enhanced classification performance by integrating machine learning with optimized algorithms. However, models based on traditional feature engineering often suffer from high complexity, limited flexibility, and the need for extensive parameter tuning (Hu et al., 2022), which hinders their scalability and practical application.

To overcome these limitations, deep learning has been increasingly adopted for hyperspectral seed analysis (Medus et al., 2021). With its capacity to extract hierarchical feature representations automatically, deep learning reduces the reliance on manual feature engineering and improves model generalization (Barbedo, 2023). Li et al. (2023) integrated the CBAM attention module into the MobileNetV3 network for corn seed defect detection, achieving 93.14% accuracy. Zhang et al. (2022) combined a convolutional autoencoder with a bionic recognition model for incremental learning, achieving 98.76% accuracy. Qi et al. (2024) applied several deep learning models—including LeNet, GoogLeNet, and ResNet—for classifying ten watermelon seed varieties, with a maximum accuracy of 99.56%. Wang et al. (2024) developed an improved E-VGG16 model by incorporating batch normalization and deep convolutional layers, achieving 98.13% accuracy. Li et al. (2024) proposed a lightweight model based on an optimized ResNet50 framework, incorporating an efficient channel attention (ECA) mechanism and depthwise separable convolutions, achieving 91.23% accuracy. Zhang et al. (2024) used a sparse attention mechanism for hyperspectral band selection, resulting in 93.27% classification accuracy. Tang et al. (2023) introduced the HSI-3DResNet model using 3D convolution to extract joint spatial–spectral features, achieving an accuracy of 97.47%.

Currently, the application of convolutional neural networks (CNNs) to hyperspectral image (HSI) processing can be broadly categorized into three primary approaches. The first approach employs dimensionality reduction or feature selection techniques—such as the Successive Projections Algorithm (SPA) and Competitive Adaptive Reweighted Sampling (CARS)—to reduce spectral dimensionality prior to classification, thereby lowering computational complexity. However, this strategy often neglects the spatial structural information that is intrinsic to HSIs. The second approach transforms high-dimensional HSI data into three-channel pseudo-RGB images using methods such as Principal Component Analysis (PCA), enabling the use of conventional 2D-CNNs. While simplifying model input, this process inevitably leads to the loss of critical spectral information, substantially limiting classification accuracy.

To overcome these limitations, a third class of methods has emerged, leveraging 3D-CNNs (e.g., HSI-3DResNet) and attention-based CNN architectures to jointly extract spatial-spectral features. These models preserve partial spectral structure and enhance focus on informative bands or regions through attention mechanisms. Nonetheless, they remain insufficient in capturing the continuous spectral dependencies across sequential HSI bands. This shortcoming is primarily attributed to the fixed-size nature of 3D convolutional kernels, which offer limited receptive fields along the spectral dimension and fail to model long-range spectral continuity. Furthermore, most attention modules are primarily designed for spatial modeling, with limited capability to explicitly learn inter-band sequential relationships. As a result, these methods often suffer from incomplete spectral feature extraction, particularly in complex classification tasks.

To address this issue, we propose a CNN architecture with variable-depth convolutional kernels, enabling multi-scale receptive fields that can flexibly adapt to different spectral correlations. By preserving the continuity of spectral signatures while simultaneously integrating spatial features, the proposed method enables efficient joint spectral-spatial modeling without relying on explicit dimensionality reduction. Experimental results demonstrate that our approach significantly enhances classification performance in high-precision tasks such as corn seed variety identification, offering a promising solution for detailed hyperspectral analysis.

## 2 Materials and methods

### 2.1 Preparation of experimental materials

The corn seeds used in this study were sourced from the corn seed bank of the Jilin Academy of Agricultural Sciences, which is known for its comprehensive collection of high-quality varieties. Eight corn seed varieties were carefully selected based on their intact appearances and overall seed quality. These varieties included Youdi919, Xianyu335, Simi21, KX3564, Ruipu909, Zhongdan111, Limin33, and Jidan209, which represent a diverse range of genetic backgrounds and characteristics commonly used in commercial production. For each variety, 100 seeds were meticulously collected, totaling 800 experimental samples. This selection process ensured that the samples were representative of the variations typically encountered in real-world agricultural settings.

The sample size of 100 seeds per variety is considered sufficiently large to provide robust data for statistical analysis and model validation. With a total of 800 seeds across eight distinct varieties, the sample size is large enough to capture a wide range of variability within and between varieties, which is crucial for drawing reliable conclusions about the generalizability of the results. This sample size ensures that the findings from this study can be confidently extended to other similar varieties, enhancing the applicability of the research to broader contexts.

**Figure 1:**
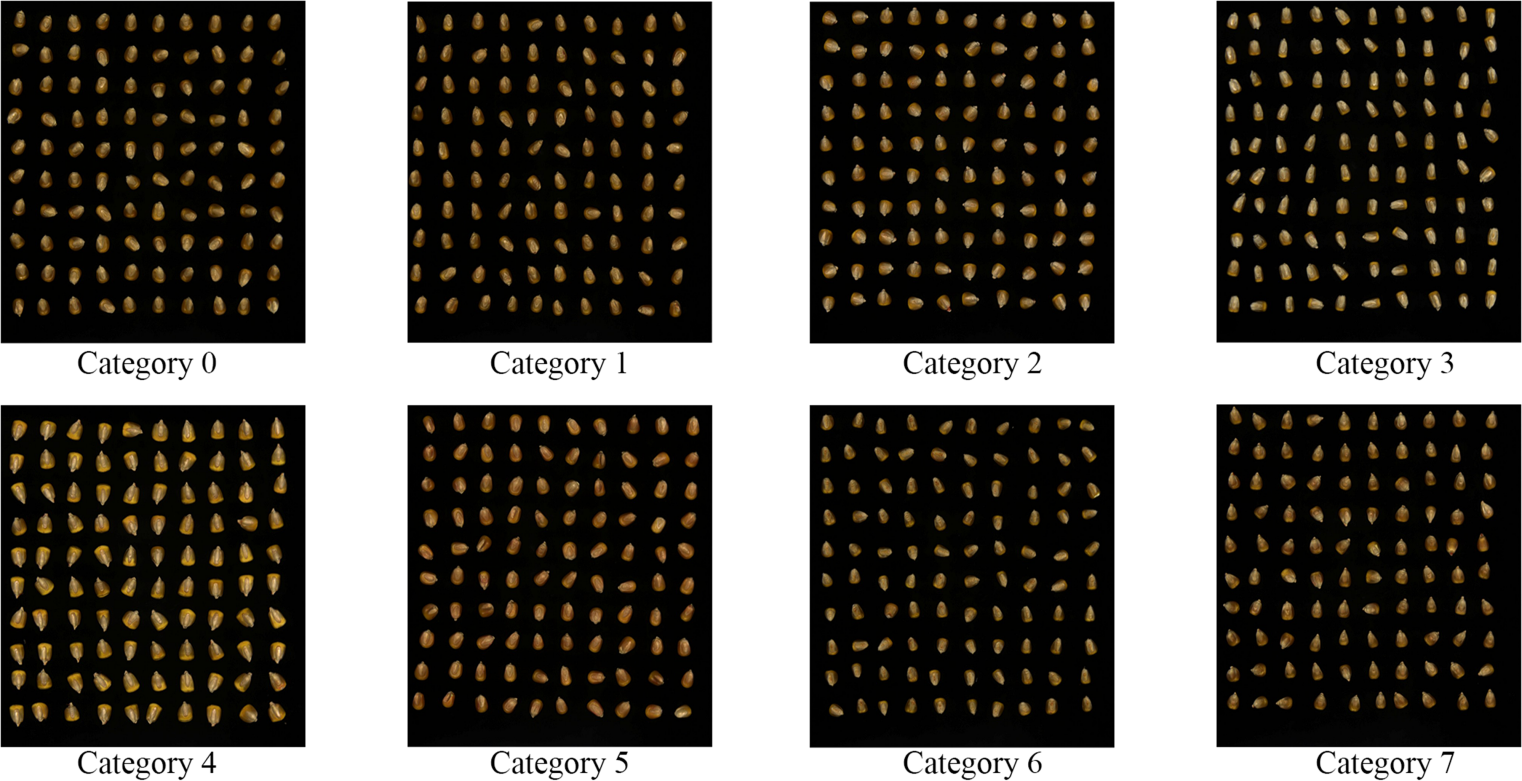
8 varieties of corn seed samples.

Moreover, the diverse selection of varieties helps address potential variability in seed traits, such as size, shape, and surface characteristics, further strengthening the reliability and applicability of the experimental results. For the purpose of easier labeling and organization during the experiment, the eight corn seed varieties were designated as Category0, Category1, Category2, Category3, Category4, Category5, Category6, and Category7. The experimental samples are visually represented in **Figure 1**.

### 2.2 Experimental equipment and environment

**Figure 2:**
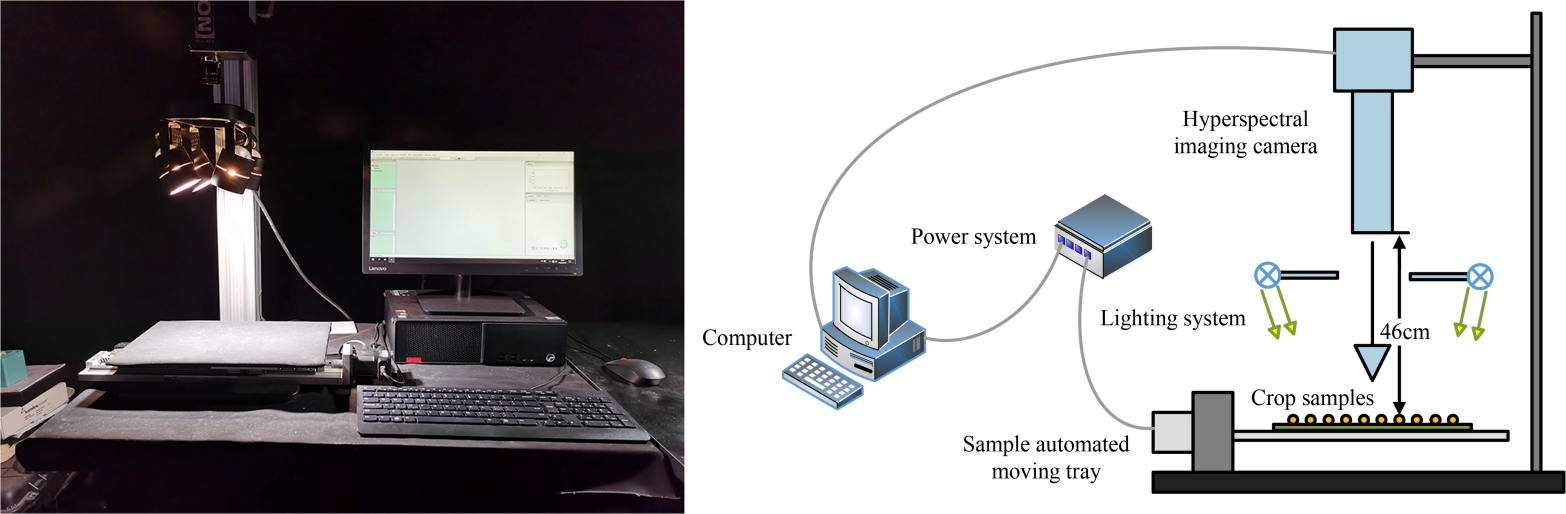
The data collection equipment consists of a Hyperspectral imaging camera, lighting system, sample automated moving tray, power system, and computer. The entire experimental process is conducted in a darkroom.

The hyperspectral imaging system used in this study comprises a hyperspectral camera (Pika XC2, Resonon Inc., Bozeman, MT, USA), a zoom lens (XENOPLAN, F/1.4 FL 23 mm, Schneider-KREUZNACH, Bad Kreuznach, Germany), a computer (Lenovo, China), a movable tray, and four halogen supplementary lights (OSRAM, Munich, Germany). The hyperspectral camera was positioned 46 cm above the tray, which measured 30 cm × 40 cm. To eliminate the influence of ambient light sources, all hyperspectral image acquisitions were performed in a darkroom. The experimental setup is illustrated in **Figure 2**.

Hyperspectral image acquisition was conducted using Resonon Pro software with the following configuration parameters: a wavelength range of 400–1000 nm, a spectral resolution of 1.3 nm, 462 spectral bands, a spatial resolution of 0.15 mm/pixel, a maximum frame rate of 165 frames per second, and a tray movement speed of 30 mm/s. Image acquisition was performed using a line-by-line scanning method.

For hyperspectral image processing and data analysis, ENVI 5.6, MATLAB R2022b, and Visual Studio Code 1.86.2 were utilized. The software environment consisted of Windows 10 22H2, Python 3.11, scikit-learn 1.4.2, PyTorch 2.2.1, and CUDA 12.1. The hardware environment included an AMD Ryzen 7 5800X 8-Core Processor (3.80 GHz), 64 GB DDR4 RAM, and an NVIDIA GeForce RTX 3090 GPU.

### 2.3 Hyperspectral image acquisition and sample segmentation

In order to reduce the impact of light source fluctuations and dark current on high-spectral images, it is necessary to perform black and white correction on the experimental equipment. Place a polytetrafluoroethylene standard white plate with a reflectance of 99.99% at the same height as the sample, scan and collect the standard white light calibration data W . Then cover the lens with a black lens cap and collect the dark field calibration data B . If the original image is $I_{0}$ , the final corrected data I is obtained as shown in Equation 1:


(1) $$I=\frac{I_{0}-B}{W-B}$$

**Figure 3:**
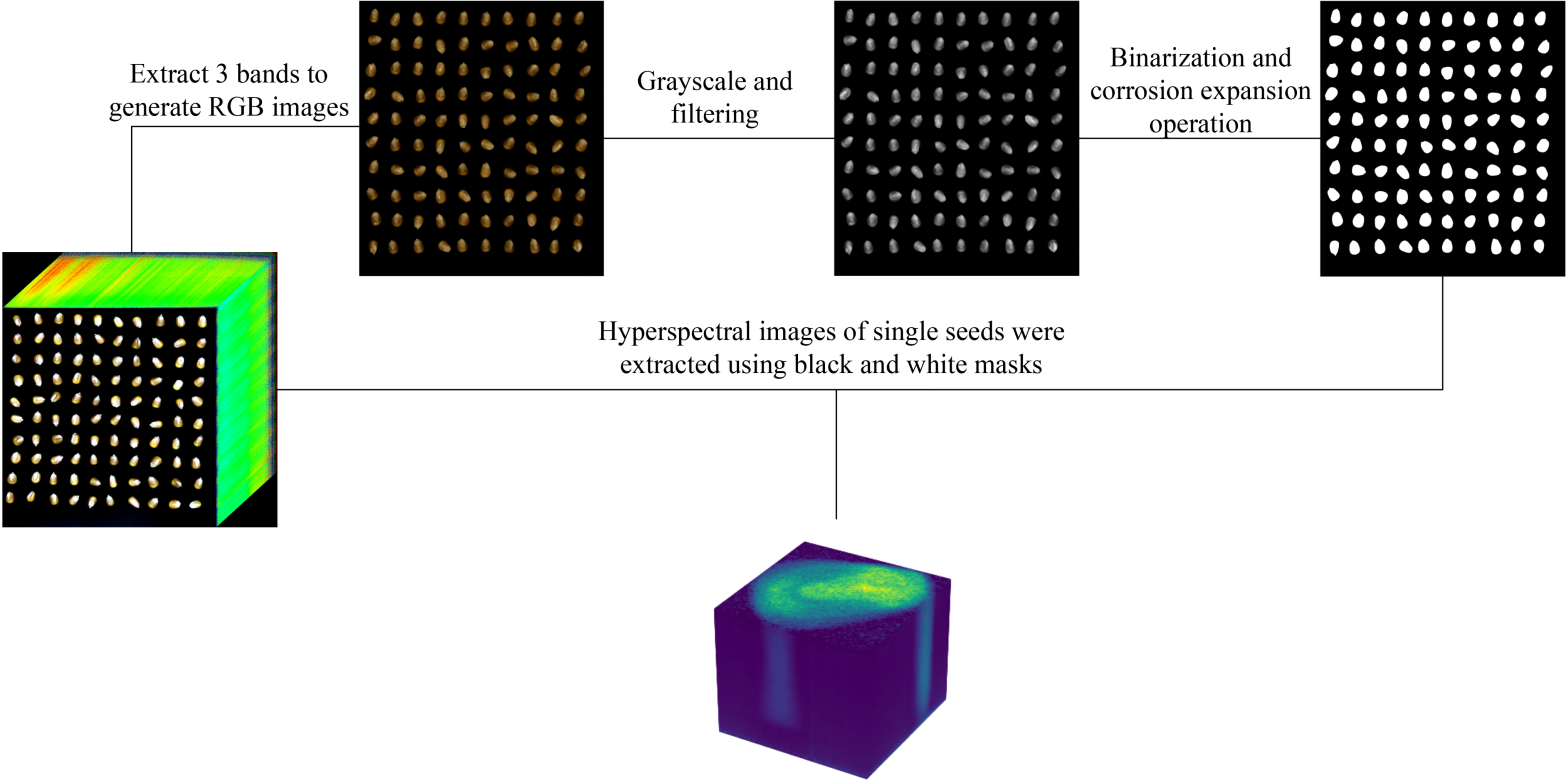
Using RGB color images processed to construct segmentation masks to extract hyperspectral image data of individual seeds.

The acquired hyperspectral images were opened using ENVI software, and a color composite image was constructed using the images at 640 nm, 550 nm, and 470 nm wavelengths, which are the default band selections in ENVI for generating RGB composites. The generated color images are then processed using a series of image processing techniques. First, the images are converted to grayscale using a weighted average method. Next, mean filtering with a 7 × 7 kernel is applied to reduce noise. Finally, binary thresholding is employed to perform threshold segmentation, generating a mask for extracting the regions of interest corresponding to individual seed samples. The hyperspectral data for each segmented seed are then extracted from the original hyperspectral dataset, forming the experimental dataset. The extraction process and results are illustrated in **Figure 3**.

**Figure 4:**
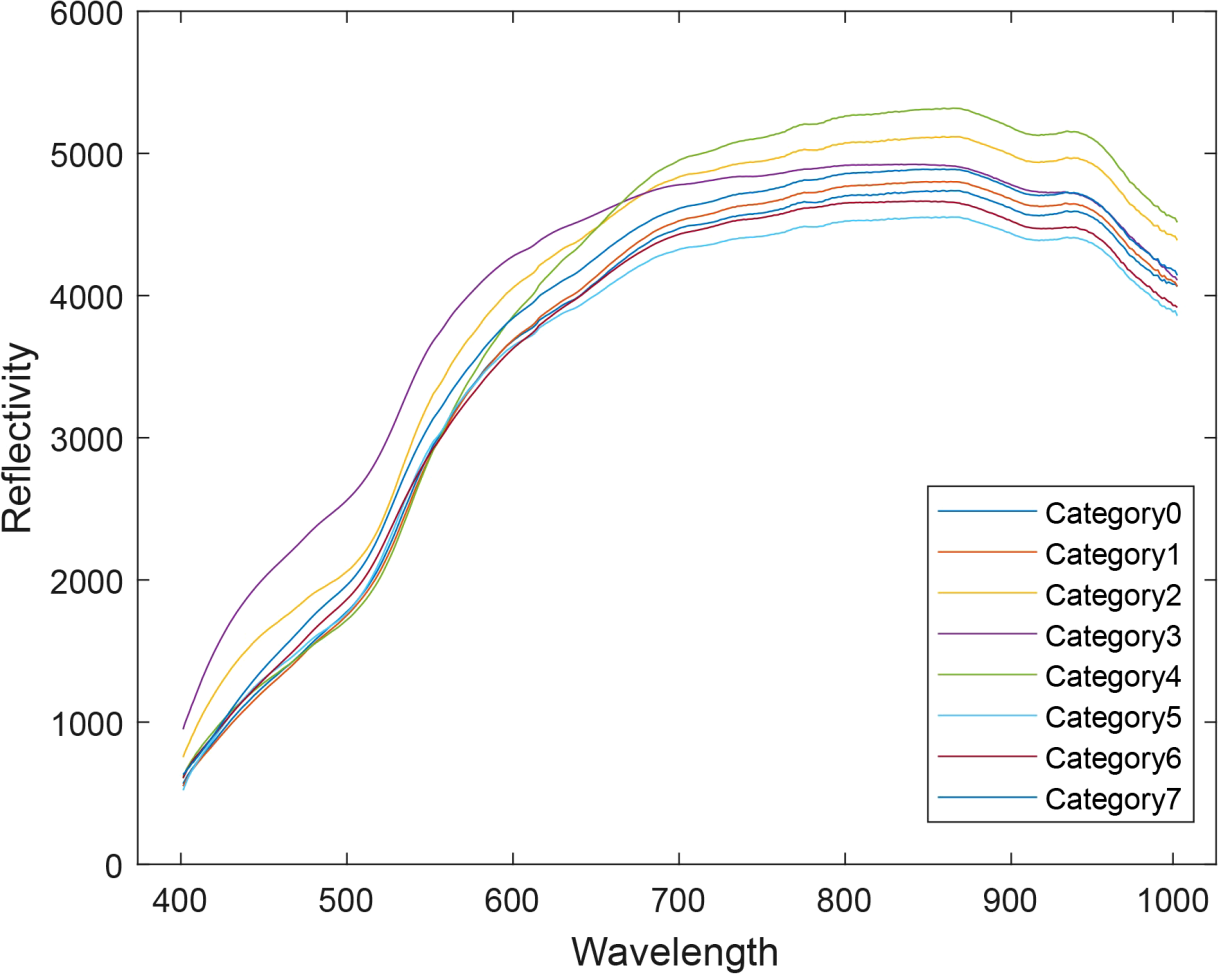
Average spectral profiles of eight corn seed varieties.

The average spectra of all samples were extracted, as shown in **Figure 4**. Significant differences in the spectral curves are observed between the wavelengths of 500 nm–550 nm and 600 nm–700 nm, with notable variations in both the shape and intensity of the curves.

**Figure 5:**
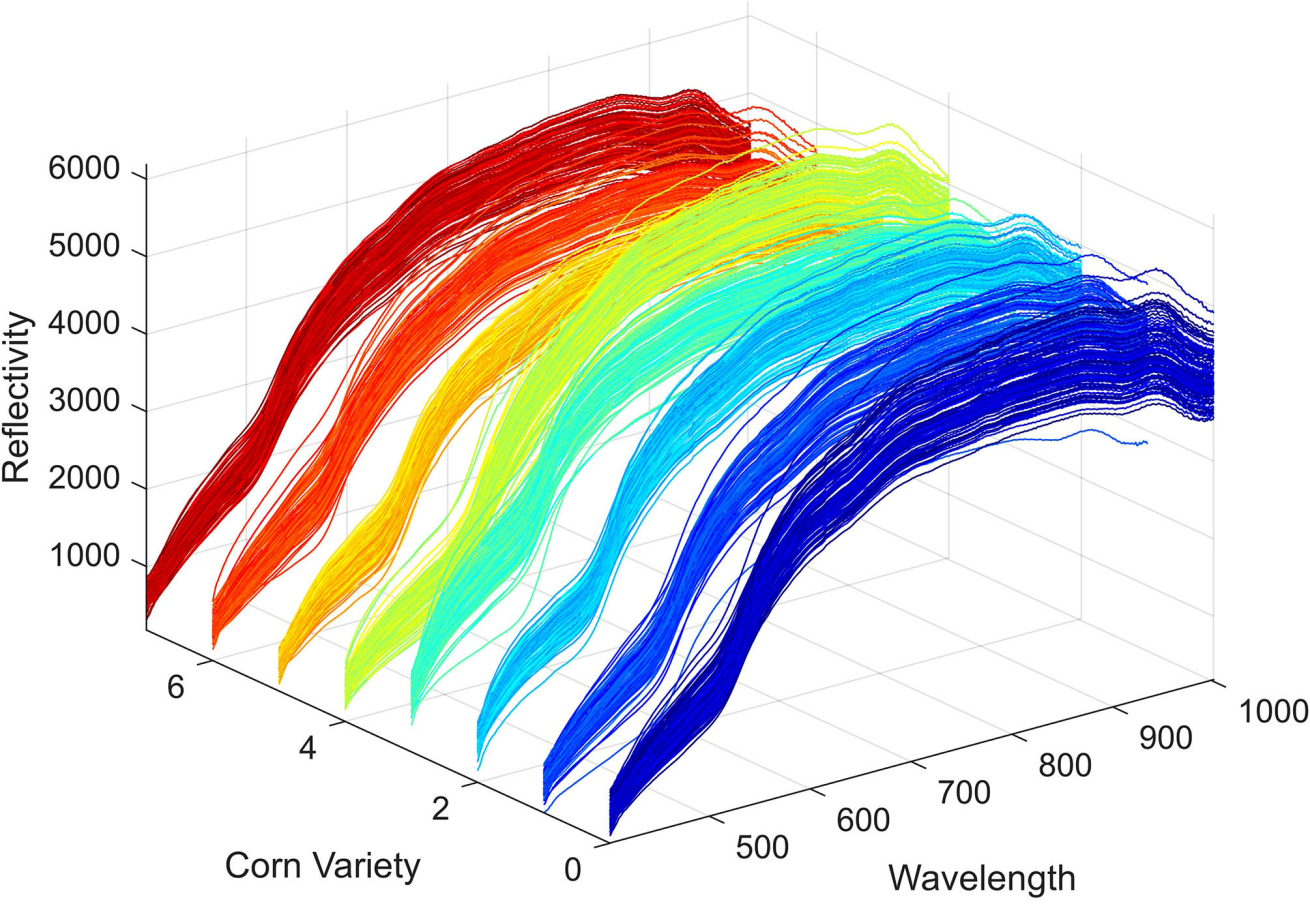
Summary of average spectral profiles for each sample.

The summary of the average spectral profiles for each sample is shown in **Figure 5**. Within the same variety, the spectral curves of each sample are generally consistent in shape, with the primary differences observed in intensity. This confirms that the spectral variation within samples of the same variety is minimal. When combined with the previous observation of significant spectral differences between varieties, it can be concluded that the hyperspectral image contains valuable variety-specific features and differential information, which serves as a foundational dataset for variety classification.

Due to the varying sizes of the extracted images, a standardization procedure is applied to the 800 experimental samples to uniformly resize each sample’s hyperspectral image to 462×100×100. In this study, the 800 experimental samples are divided into training and testing sets in a 4:1 ratio, with 640 samples in the training set and 160 samples in the testing set. The recognition accuracy for both the training and testing sets is calculated and analyzed separately.

## 3 Model construction

### 3.1 Construction of variable-depth convolutional kernels

Hyperspectral data is a three-dimensional structured dataset consisting of multiple spectral bands, which not only captures spectral feature information but also incorporates spatial texture characteristics of the image. Due to its unique 3D structure, hyperspectral data exhibits rich features in both the spatial and spectral dimensions, posing challenges for conventional convolutional kernels in processing such data. The three-dimensional nature of hyperspectral data requires convolutional kernels to simultaneously extract feature information across all three dimensions.

To address the limitations of traditional 3D convolutional kernels in hyperspectral data analysis, a Variable-Depth Convolutional Kernel (VD) structure is proposed. This structure is designed to extract both spectral and texture features from hyperspectral data.

The core concept of the variable-depth convolutional kernel is based on the understanding that different datasets or regions may exhibit varying degrees of correlation in the depth dimension. Traditional fixed-depth convolutional kernels may limit the model’s feature extraction capabilities. Therefore, dynamic adjustment in the depth dimension is introduced, allowing the model to adaptively select the appropriate depth range based on local data characteristics. This approach enables the extraction of meaningful features at multiple depth scales.

**Figure 6:**
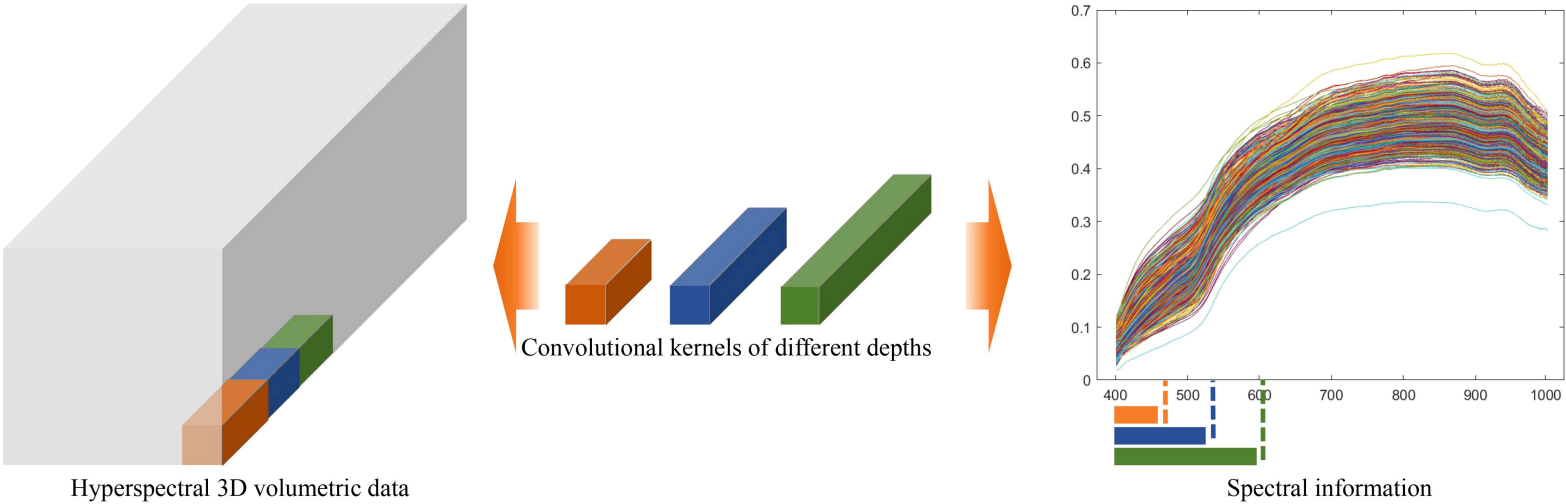
Schematic diagram illustrating the feature extraction differences of convolutional kernels with varying depths in hyperspectral data.

As illustrated in **Figure 6**, the computational effects of 3D convolutional kernels with varying depths on hyperspectral structured data demonstrate that, as the depth of the convolutional kernel increases, it can extract more comprehensive spectral feature information. This further highlights the advantages of the variable-depth convolutional kernel in hyperspectral data processing.

**Figure 7:**
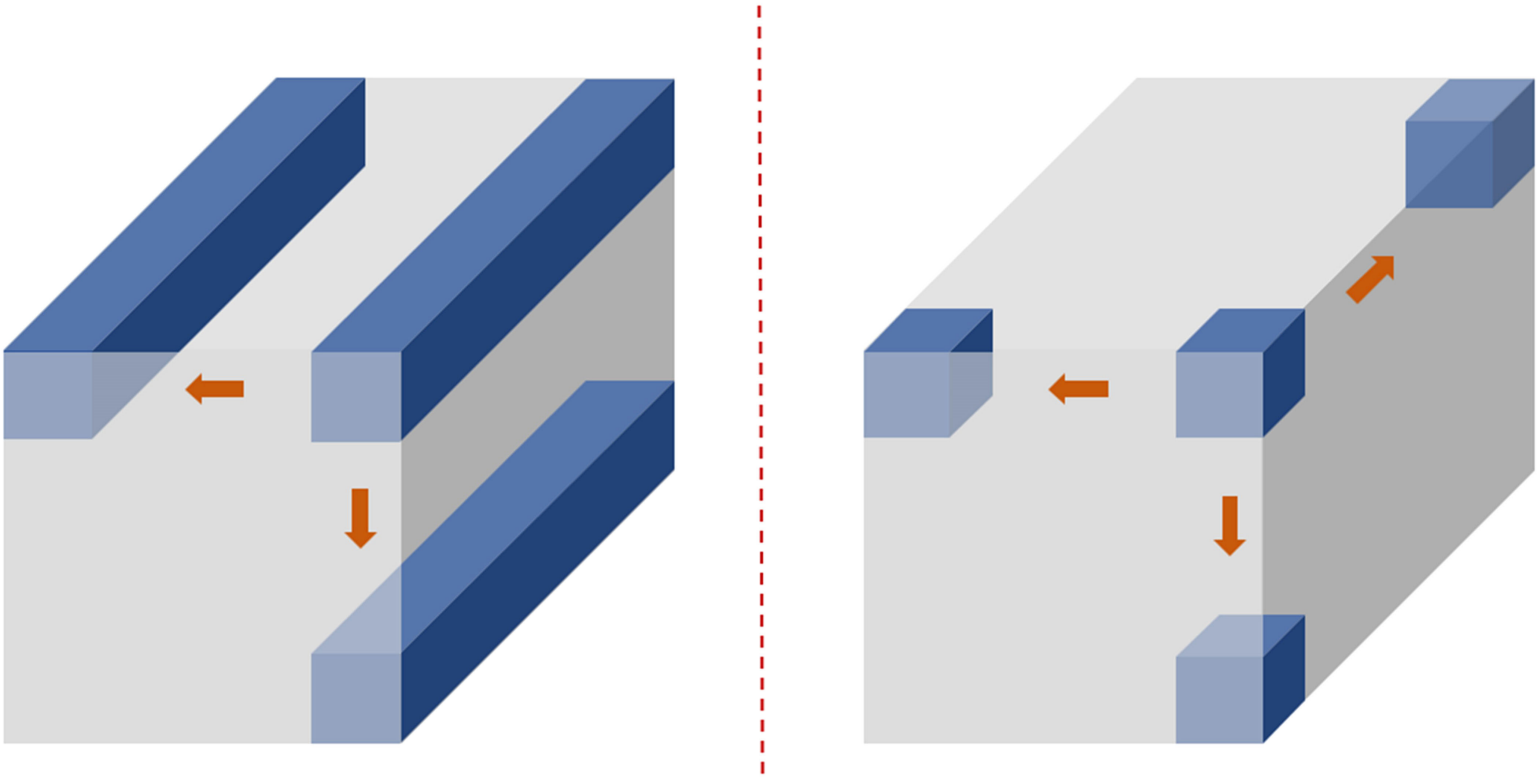
Schematic diagram of the 2D and 3D convolution operation processes.

There are significant differences between two-dimensional convolutional neural networks (2D CNNs) and three-dimensional convolutional neural networks (3D CNNs) in terms of convolution operation dimensions, data structure processing, computational complexity, and application scenarios. The differences in their computational processes are illustrated in **Figure 7**. In the 2D convolution operation, sliding occurs only within the two-dimensional plane, and the feature extraction is completed by fully covering all the plane information. In contrast, the 3D convolution operation requires sliding not only within the two-dimensional plane but also along the depth dimension. Compared to 2D convolution, 3D convolution can handle more complex structural data and extract more feature information.

In three-dimensional convolution operations, the input data is represented as a three-dimensional tensor, typically denoted as $X\in R^{H*W*D}$ , where H is the height, W is the width, and D is the depth of the input data. The three-dimensional convolutional kernel K is a three-dimensional tensor, generally represented as $K\in R^{F*F*D_{k}}$ , where F is the two-dimensional spatial dimension of the kernel, and $D_{k}$ is the depth of the kernel. The standard three-dimensional convolution operation is described by the following Equation 2. The value at a specific position in the output feature map of a 3D convolution operation can be expressed as follows:


(2) $$y\;\left(i,j,k\right)=\sum_{m=0}^{F-1}\sum_{n=0}^{F-1}\sum_{p=0}^{D_{k}-1}X\left(i+m,j+n,k+p\right)\cdot K\left(m,n,p\right)$$

**Figure 8:**
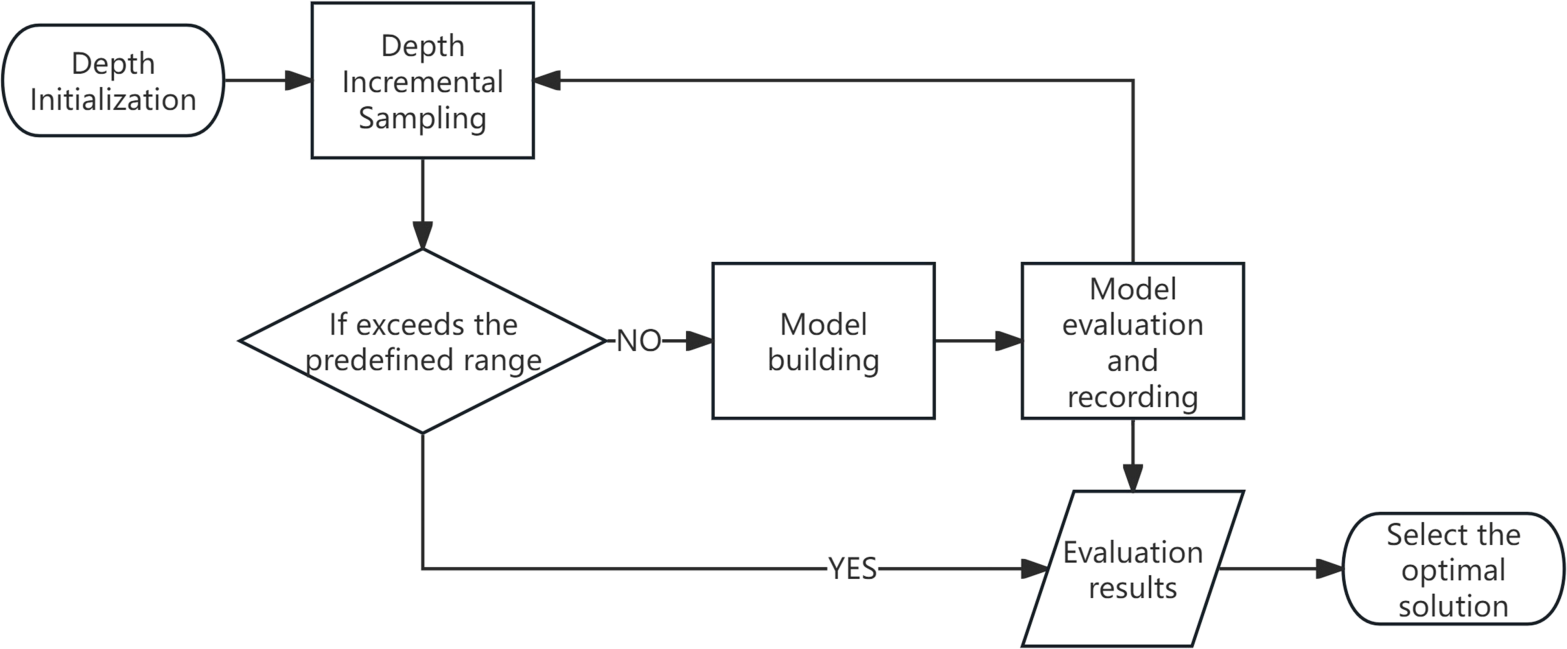
Flow diagram of the optimal depth determination process for variable-depth convolutional kernels.

This study draws conceptual inspiration from the Successive Projections Algorithm (SPA), which identifies characteristic wavelengths through a process of variable initialization, iterative refinement, and performance-based selection of the optimal solution (de Souza et al., 2025). In a similar manner, the proposed variable-depth convolutional kernel is constructed through an iterative optimization strategy, as schematically illustrated in **Figure 8**, wherein the kernel depth is dynamically adjusted and refined based on performance evaluations throughout the training process. The fundamental principle involves dynamically adjusting the depth of the convolutional kernel at each iteration, evaluating the resulting model performance, and ultimately selecting the configuration that yields the highest classification accuracy.

The process begins by defining a feasible range for the kernel depth parameter. In the context of hyperspectral data analysis, where the informative spectral regions are typically confined to specific bands, selecting an excessively broad initial depth range may introduce spectral redundancy and impair feature extraction efficiency. To mitigate this, the initial range is carefully bounded to balance expressiveness and computational cost.

Following initialization, the kernel depth is incrementally sampled within the predefined range. At each sampling step, a corresponding convolutional neural network model is instantiated, trained on the available data, and quantitatively evaluated using appropriate performance metrics (e.g., overall accuracy or F1-score). The evaluation results are recorded for each depth setting. This iterative procedure continues until the upper bound of the kernel depth range is reached.

After all candidate configurations have been assessed, the depth value that achieves the best classification performance is selected as the optimal setting for the convolutional kernel. This adaptive mechanism enables the network to self-tune its architectural complexity based on data-driven performance feedback, thereby enhancing its capacity for efficient and discriminative feature representation in hyperspectral image analysis.

First, define $P(D_{k})$ as the model’s accuracy when using a convolutional kernel with a depth of $D_{k}$ , and let $D_{max}$ represent the maximum depth of the convolutional kernel. Based on the spectral characteristics of hyperspectral data, the typical response length of continuous bands ranges from 10 to 20. Therefore, $D_{max}$ is set to 25 as the termination criterion for the computation process. $D_{best}$ denotes the optimal convolutional kernel depth. Next, by continuously increasing the convolutional kernel depth $D_{k}$ , the optimal depth $D_{best}$ is determined, which maximizes the accuracy $P(D_{k})$ . The specific process is shown in Equation 3.


(3) $$maximize\;P(D_{k})\;with\;respect\;to\;D_{k}$$

The variation in convolutional kernel depth is shown in Equation 4.


(4) $$D_{k}^{\left(t+1\right)}=D_{k}^{\left(t\right)}+2$$

The process by which the variable-depth convolutional kernel maximizes the model accuracy by incrementally increasing the convolutional kernel depth can be expressed by Equation 5.


(5) $$\;D_{best}=arg_{1\leq D_{k}\leq D_{max}}maxP\left(D_{k}\right)$$

Due to the dynamic nature of the variable-depth convolutional kernel, whose depth changes with each iteration, conventional 3D convolutional neural networks with fixed kernel depth cannot be directly applied to this structure. To accommodate the depth variability of the convolutional kernel throughout the iterative process, a novel convolutional neural network architecture must be designed. This model should not only support the computational mechanism of variable-depth kernels but also incorporate necessary modifications in network architecture, parameter optimization, and feature extraction strategies. These adaptations are essential to fully exploit the adaptive depth capability of the kernels, thereby enabling efficient modeling and classification of complex data structures.

### 3.2 Establishment of a variable-depth convolutional neural network

In this study, a corn seed variety recognition model is proposed and implemented based on the PyTorch framework. The core architecture consists of an input layer, variable-depth (VD) convolutional layers, max-pooling layers, fully connected layers, and dropout layers. The input layer receives three-dimensional hyperspectral data with dimensions of 462×100×100.

**Figure 9:**
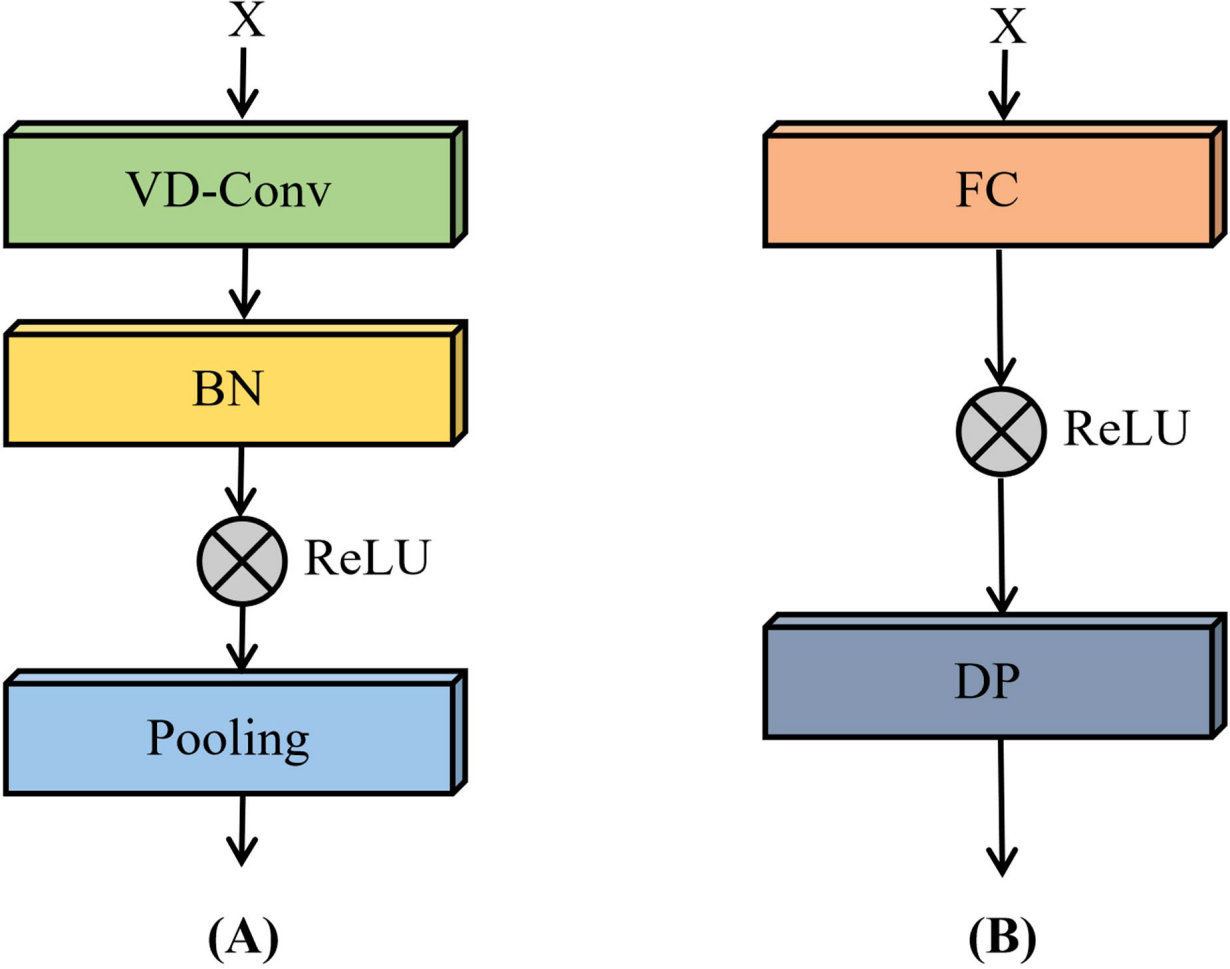
Structure of the convolution module **(A)** and fully connected module **(B)** in the VD-CNN.

The VD convolutional module, as illustrated in **Figure 9A**, is composed of VD convolutional layers, batch normalization layers, ReLU activation functions, and max-pooling layers. In this module, the VD convolutional layers are designed to adaptively extract both textural and spectral features. Batch normalization facilitates faster convergence during training and improves the stability of the model. The ReLU activation function introduces non-linearity, enabling the network to capture complex interactions among features. Max-pooling layers are subsequently employed to reduce spatial dimensionality and suppress redundant information, thereby enhancing computational efficiency and improving generalization performance.

The fully connected module, shown in **Figure 9B**, integrates fully connected layers, ReLU activation functions, and dropout layers. In this module, the fully connected layers transform and consolidate high-level abstract features extracted by the preceding VD convolutional module. Dropout layers are incorporated as a regularization technique to mitigate overfitting by randomly deactivating a subset of neurons during training, ultimately contributing to improved model generalizability.

**Figure 10:**
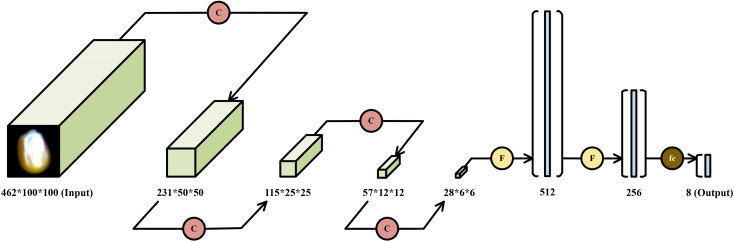
Architecture diagram of the VD-CNN model.

Ultimately, the proposed VD-CNN model is constructed by sequentially stacking four VD convolutional modules and three fully connected modules. The overall architecture of the model is depicted in **Figure 10**. Each VD convolutional module is designed to progressively extract multi-scale spectral and spatial features, while the fully connected modules are responsible for integrating these high-level features and performing final classification.

In this research, the depth parameter range is set between 3 and 25. To facilitate a comparative analysis of the impact of different kernel depths on model classification accuracy, all depth values generated during the depth selection process were manually extracted, and corresponding VD-CNN models were constructed for each depth configuration. The width and height of the kernels are fixed at 3×3, while the depth of the kernels, denoted as d, ranges from 3 to 25 in increments of 2 (i.e., 3, 5, 7, 9, 11, 13, 15, 17, 19, 21, 23, and 25), resulting in 12 different VD kernel sizes. The kernel size for the remaining convolutional layers is set to 3×3×3. Since each band in the hyperspectral images represents one-dimensional data, the number of convolution operation channels for all convolutional layers is set to 1. The padding size for the first and second convolutional layers is set to half the size of the kernel, while the padding for the remaining convolutional layers is set to 1. The parameters for the fully connected layers are configured as (1008, 512), (512, 256), and (256, 8), and the dropout layer parameter p is set to 0.5. A comparison of the model parameters is shown in **Table 1**.

**Table 1:** The comparison information of the model parameters.

Model Name	Kernel size			Padding size	Fully connected layer1	Fully connected layer2	Fully connected layer3
	w	h	d				
M3	3	3	3	1	(1008, 512)	(512, 256)	(256, 8)
M5	3	3	5	2	(1008, 512)	(512, 256)	(256, 8)
M7	3	3	7	3	(1008, 512)	(512, 256)	(256, 8)
M9	3	3	9	4	(1008, 512)	(512, 256)	(256, 8)
M11	3	3	11	5	(1008, 512)	(512, 256)	(256, 8)
M13	3	3	13	6	(1008, 512)	(512, 256)	(256, 8)
M15	3	3	15	7	(1008, 512)	(512, 256)	(256, 8)
M17	3	3	17	8	(1008, 512)	(512, 256)	(256, 8)
M19	3	3	19	9	(1008, 512)	(512, 256)	(256, 8)
M21	3	3	21	10	(1008, 512)	(512, 256)	(256, 8)
M23	3	3	23	11	(1008, 512)	(512, 256)	(256, 8)
M25	3	3	25	12	(1008, 512)	(512, 256)	(256, 8)

### 3.3 Optimizer and loss function

This study utilizes the Adam optimizer to optimize the model’s learning rate, with the parameter lr set to 0.001. The Adam optimizer combines the advantages of Adagrad and RMSprop, dynamically adjusting the learning rate of each parameter based on the first and second moment estimates of the gradient of each parameter, thereby achieving a better balance between convergence speed and stability during the training process (Kingma and Ba, 2014). The specific formula for the update rule of the Adam optimization algorithm is shown in Equations 6-10. And m and v represent the first and second moment estimates, respectively, g denotes the gradient of the current parameter, $\beta_{1}$ and $\beta_{2}$ are exponential decay rates, t represents the current iteration number, $\hat{m}$ and $\hat{v}$ represent the corrected first and second moment estimates, θ represents the model parameters, α is the learning rate, ϵ is a very small number used for numerical stability.


(6) $$m=\beta_{1}\cdot m+\left(1-\beta_{1}\right)\cdot g$$

(7) $$v=\beta_{2}\cdot v+\left(1-\beta_{2}\right)\cdot g^{2}$$

(8) $$\hat{m}=\frac{m}{1-\beta_{1}^{t}}$$

(9) $$\hat{v}=\frac{v}{1-\beta_{2}^{t}}$$

(10) $$\theta =\theta -\frac{\alpha }{\sqrt{\hat{v}}+\epsilon }\cdot \hat{m}$$

This study employs the cross-entropy loss function to train the model, specifically for multi-classification problems, with the specific formula shown in Equation 11. N represents the number of classes, $y_{i}$ is the label for the i th class, and ${\hat{y}}_{i}$ is the predicted probability output by the model. The cross-entropy loss function is a commonly used loss function for classification problems, especially widely applied in deep learning. In binary or multi-class tasks, the cross-entropy loss function can measure the disparity between the predicted probability distribution of the model output and the actual labels (Mao et al., 2023).


(11) $$L\left(y,\hat{y}\right)=-\sum_{i=1}^{N}y_{i}log\left({\hat{y}}_{i}\right)$$

### 3.4 Evaluation metrics

In this study, the overall evaluation of the model is conducted by recording the loss value, training time, accuracy, recall, precision, and F1 score of both the training and testing sets for each epoch. The specific calculation formulas are shown in Equations 12-15. In this case, TP, TN, FP, and FN represent the true positive, true negative, false positive, and false negative values of the confusion matrix, respectively. The main evaluation metric is based on the F1 score, indicating a good balance between precision and recall, signifying accurate and comprehensive classification performance of the model.


(12) $$Accuracy=\frac{TP+TN}{TP+TN+FP+FN}$$

(13) $$Recall=\frac{TP}{TP+FN}$$

(14) $$Precision=\frac{TP}{TP+FP}$$

(15) $$F1\;Score=2\times \frac{Precision\times Recall}{Precision+Recall}$$

## 4 Results

**Figure 11:**
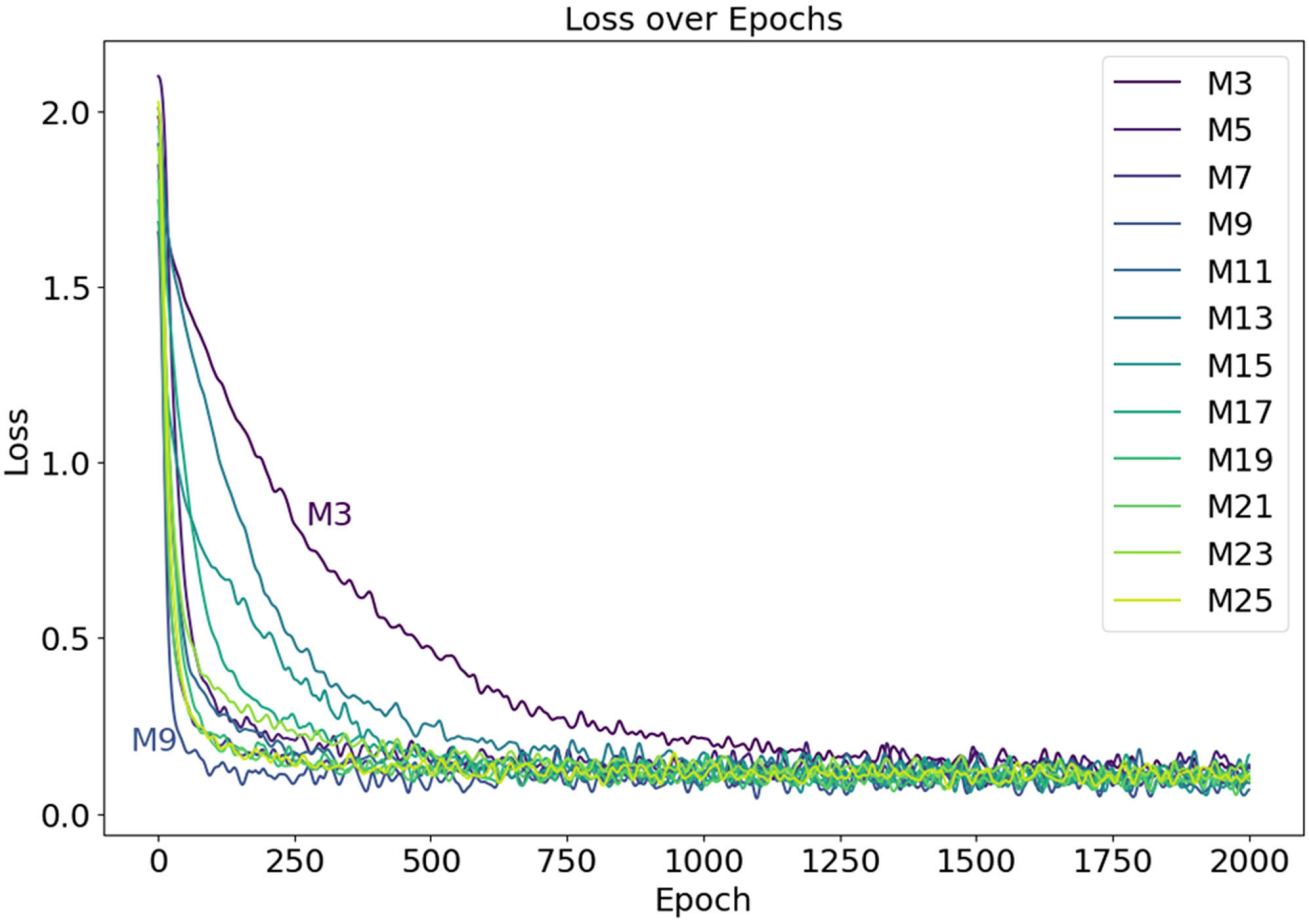
The Loss curves during the model training process, with the curves of models M3 and M9 labeled in the figure.

The neural network models, distinguished by varying depths of convolutional kernels, are labeled as M3, M5, M7, M9, M11, M13, M15, M17, M19, M21, M23, and M25, based on the depth of the convolutional kernels used. The experimental outcomes of these models, utilizing 12 distinct convolutional kernel depths, are carefully analyzed, with the reduction curves of loss values presented in **Figure 11**. It is evident that the model labeled M3, using 3×3×3 convolutional kernels, exhibits the slowest convergence speed, with the loss plateauing between 0.1 and 0.2 only after 1500 iterations. Additionally, an analysis of the fluctuating curves reveals that the convergence speeds of the other 11 models surpass that of M3, with the model M9 demonstrating the fastest convergence among the group.

**Figure 12:**
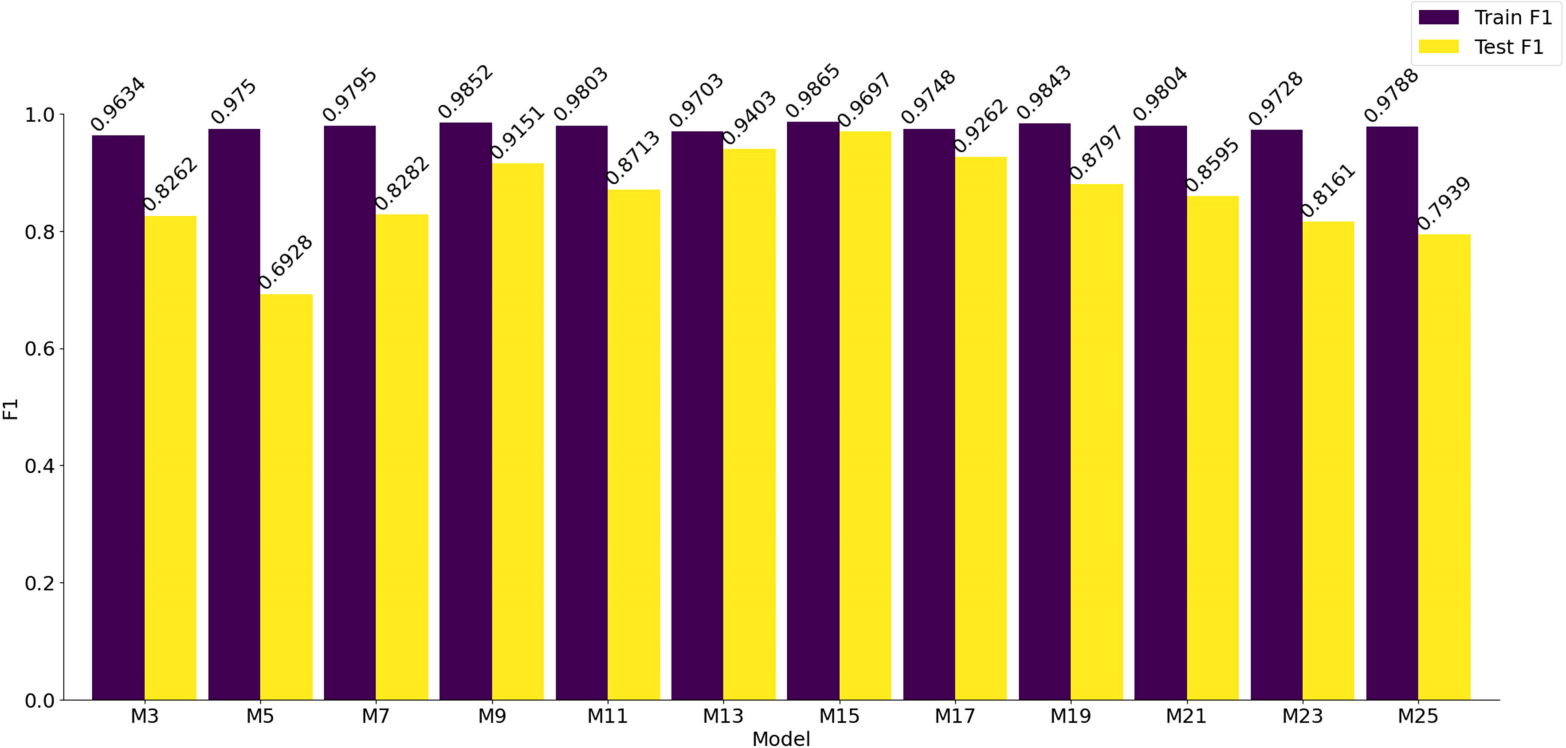
Statistical chart of the F1 scores on the training set and the testing set of the model.

The bar chart displaying the F1 scores of the models on both the training and testing sets is shown in **Figure 12**. It is evident that the F1 scores of all models on the training set are approximately 0.97, with the highest score achieved by the model labeled M15, which attains an F1 score of 0.9865. Furthermore, when analyzing the F1 scores on the testing set, M15 also achieves the highest score of 0.9697. The trend of F1 score variations demonstrates a gradual increase from M3, reaching its peak at M15, and then decreasing thereafter. Based on these experimental results, it can be concluded that the model labeled M15 delivers the best performance, excelling not only in training effectiveness but also in generalization capabilities.

**Figure 13:**
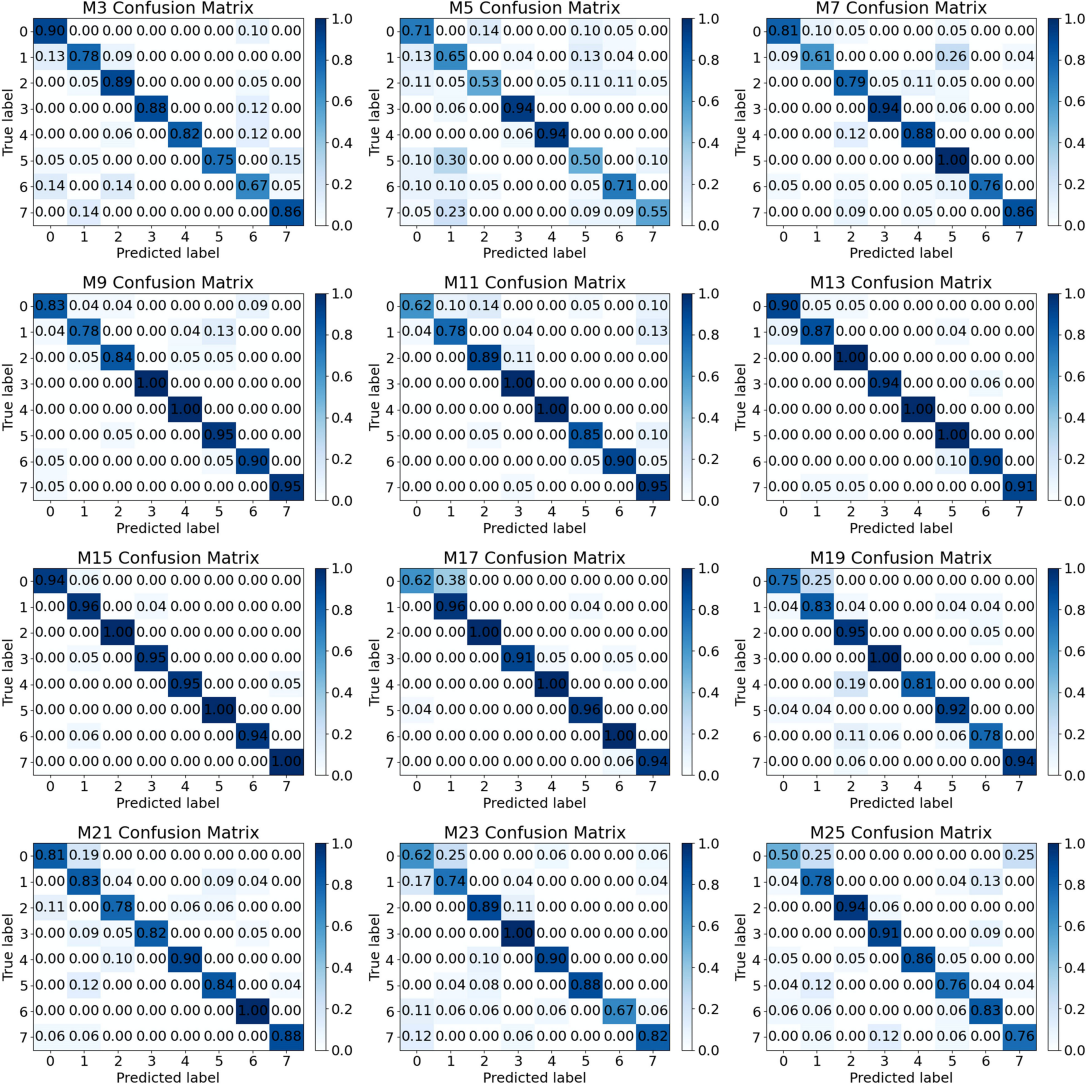
Confusion matrix of the model on the test set.

The confusion matrix for the model’s testing set is shown in **Figure 13**. Notably, the model labeled M15 demonstrates the best classification performance, with all eight types of samples achieving a classification accuracy of approximately 0.95. Furthermore, the samples labeled as 2, 5, and 7 exhibit perfect classification accuracy, reaching a score of 1.

To compare the classification performance of the VD-CNN model with traditional models and to assess the impact of the VD convolution kernel, this study conducted ablation experiments using widely adopted models such as ResNet-50, VGG16, and AlexNet. The experimental results are presented in **Table 2**. It is evident that before the introduction of the VD convolution kernel, the classification performance of the traditional models was generally superior to that of the CNN model, with a classification accuracy exceeding by at least 14.14%. However, after incorporating the VD convolution kernel, the classification accuracy of the VD-CNN model reached its peak, achieving an accuracy of 96.88%.

**Table 2:** The results of the ablation experiment.

VD	CNN	ResNet-50	VGG16	AlexNet	Accuracy	Precision	Recall	F1 Score
				✓	87.64%	88.31%	87.22%	86.79%
			✓		91.32%	90.54%	92.01%	91.41%
		✓			92.5%	92.72%	92.5%	92.56%
	✓				73.5%	71.43%	72.49%	73.49%
✓	✓				96.88%	97.24%	96.82%	96.97%

As shown in **Table 3**, this comparative evaluation of several convolutional neural network architectures highlights distinct trade-offs among computational complexity, model size, and inference efficiency. VGG16 exhibits the highest computational burden, with 15.52 giga multiply-accumulate operations and 138.36 million parameters, resulting in a moderate inference time of 1.62 milliseconds per sample. Although ResNet-50 significantly reduces both parameter count and computational load—25.56 million parameters and 4.13 giga operations—its inference time increases to 5.26 milliseconds per sample, likely due to the additional overhead introduced by its deep residual connections. AlexNet achieves the fastest inference speed of 0.52 milliseconds per sample, with a moderate complexity of 716.44 million operations and 61.1 million parameters. Notably, the VD-CNN model demonstrates a compelling balance between efficiency and performance. With only 650.73 thousand parameters and a computational cost of 679.05 million operations, VD-CNN achieves a competitive inference time of 0.586 milliseconds per sample, closely matching that of AlexNet, despite having a model size nearly 100 times smaller. Compared to other architectures, VD-CNN offers superior efficiency in terms of parameter utilization and inference latency, making it particularly well-suited for deployment in resource-constrained or real-time applications. These results underscore the value of compact and well-optimized architectures like VD-CNN when balancing accuracy, latency, and hardware limitations in practical scenarios.

**Table 3:** Computational complexity, parameter size, and inference performance of different convolutional neural network architectures.

Model	FLOPs	Params	Average Inference Time
ResNet-50	4.13G	25.56M	5.26
VGG16	15.52G	138.36M	1.62
AlexNet	716.44M	61.1M	0.52
CNN	180.09M	650.63K	0.6
VD-CNN	679.05M	650.73K	0.586

To validate the applicability of the model, experiments were conducted using the publicly available hyperspectral rice seed dataset RVHID90 from the research data open platform Zenodo (Vu et al., 2019). The dataset contains 90 varieties of rice seeds, with 96 samples (seeds) per variety. Samples of each variety are imaged in two bundles of 48 seeds, arranged in a 6 by 8 grid on a black background plate. Imaging is performed simultaneously using both an RGB camera and a hyperspectral camera. The RGB camera has a resolution of 4896 by 3264 pixels. The hyperspectral camera used is a VIS/NIR (visible/near-infrared) camera, capturing reflectance across the range from 385 nm to 1000 nm over 256 discrete wavelengths. By comparing the classification models (Filipović et al., 2021; Taheri et al., 2024) from previous studies that used this dataset, the results shown in **Table 4** indicate that the proposed VD-CNN model achieves the highest classification accuracy. This demonstrates that the variable-depth convolutional kernel structure significantly enhances the completeness of feature extraction for general hyperspectral data, making a notable contribution to the development of hyperspectral classification technologies.

**Table 4:** Comparison experimental results table using the RVHID90 dataset.

Model	Accuracy	Recall	F1 Score
baseline, original 6 morphological features + spectral mean LDA (Vu et al., 2019)	79.64%	78.80%	78.27%
morphological feature set + spectral mean + NCA (Vu et al., 2019)	84.33%	83.75%	83.43%
morphological feature set + spectral mean + spectral variance + NCA (Vu et al., 2019)	86.21%	86.00%	85.65%
ResNet-50 (Filipović et al., 2021)	92.73%	92.94%	92.64%
MobileNet (Filipović et al., 2021)	88.98%	88.58%	88.52%
DenseNet121 (Filipović et al., 2021)	92.23%	91.94%	91.88%
InceptionV3 (Filipović et al., 2021)	90.96%	90.31%	90.31%
Cutom CNN (Filipović et al., 2021)	84.56%	84.04%	83.78%
ResNet-50+MobileNet+DenseNet121+InceptionV3+Cutom CNN (Filipović et al., 2021)	94.64%	94.24%	94.22%
VD-CNN	97.78%	97.81%	97.67%

## 5 Discussion and conclusion

In this study, we focused on analyzing eight different types of corn seed samples to investigate how varying depths of convolutional kernels affect the classification accuracy of VD convolutional neural networks. The results demonstrate that increasing the depth of convolutional kernels in VD convolutional neural networks can effectively reduce the accuracy loss often associated with feature extraction and dimensionality reduction processes when handling hyperspectral data of corn seeds across multiple bands. By comparing the performance of a traditional 3×3×3 convolutional kernel to that of a 3×3×15 convolutional kernel, significant improvements were observed. The F1 score for the training set increased from 0.9634 to 0.9865, and the testing set F1 score rose from 0.8262 to 0.9697. These findings indicate that increasing the depth of convolutional kernels enhances the extraction of spectral features from hyperspectral data, thereby improving classification accuracy. However, further increases in depth may lead to redundancy in the spectral feature information across consecutive bands, ultimately resulting in a decline in classification performance.

Compared with conventional CNN-based approaches in hyperspectral image (HSI) processing—such as 3D-CNNs and attention-enhanced networks—the proposed variable-depth convolutional kernel (VD-CNN) architecture exhibits several key advantages.

Traditional 3D-CNN methods rely on fixed-size convolutional kernels to jointly extract spatial-spectral features. While these architectures can partially preserve the spectral structure of HSI data, their fixed receptive fields limit the ability to adaptively capture varying spectral dependencies, especially in cases where meaningful information spans across long-range or non-uniform spectral bands. Furthermore, most attention-based mechanisms focus primarily on enhancing spatial features or selecting informative bands, yet often lack explicit modeling of spectral continuity, which is critical in high-dimensional HSI data.

In contrast, the VD-CNN introduces convolutional kernels with variable depth along the spectral axis, enabling flexible modeling of both local and global spectral dependencies. This design allows the network to dynamically adjust its receptive field based on the characteristics of the spectral data, thus capturing both fine-grained and long-range spectral relationships more effectively. Moreover, the variable-depth design supports a multi-scale feature extraction paradigm, which enhances the network’s ability to learn hierarchical spectral representations without relying on external dimensionality reduction.

Hyperspectral imaging offers a higher level of information than multispectral imaging, owing to its ability to capture continuous spectral data across a broad range of wavelengths. This increased spectral resolution enables the detection of subtle variations in the composition and characteristics of seeds, thereby enhancing the accuracy of classification and analysis. However, the complexity of hyperspectral data presents significant challenges, particularly in terms of data processing and analysis. Hyperspectral data typically requires more sophisticated algorithms, greater computational resources, and longer processing times compared to multispectral data.

In contrast, multispectral imaging captures data at a limited number of discrete wavelengths, making it more straightforward to handle and process. Although it may not provide the same level of detailed spectral information as hyperspectral imaging, multispectral imaging remains adequate for many practical applications, such as seed classification, with the added benefits of lower equipment and computational costs.

In summary, the following conclusions can be drawn:

1. Traditional methods for processing hyperspectral data, such as dimensionality reduction and feature extraction, may result in the loss of important information. The spectral information contained in consecutive bands often includes key feature details. Experimental results demonstrate that, when using the traditional 3×3×3 convolutional kernel size, increasing the kernel size significantly improves classification accuracy. However, traditional three-dimensional convolutional neural networks are insufficient for effectively handling these compressed features.
2. By adjusting the depth of convolutional kernels in a VD convolutional neural network, the model’s classification accuracy and generalization performance can be significantly enhanced. However, surpassing the optimal depth threshold for consecutive feature bands can lead to a decline in both classification accuracy and generalization performance.
3. The introduction of the VD convolution kernel effectively improves the model’s classification accuracy. Experimental results show that the CNN model’s classification accuracy increased from 73.5% to 96.88%, demonstrating the importance of simultaneously extracting both texture features and continuous spectral features from hyperspectral data.
4. The experimental findings of this study indicate that, for the dataset under consideration, a convolutional kernel depth of 15 achieved a training accuracy of 0.9865 and a testing accuracy of 0.9697. At this depth, the classification performance was optimal, enabling rapid, effective, and non-destructive classification of corn seeds.
5. The variable-depth convolutional kernel structure can not only be applied to the corn seed hyperspectral image dataset, but also effectively extract features from general hyperspectral image data, providing a new technological approach for the development of hyperspectral technology.

The findings of this study are primarily aimed at agricultural researchers, seed producers, farmers, and related equipment manufacturers. The outcomes of this research are significant for advancing agricultural modernization, supporting the protection and breeding of germplasm resources, and offering valuable insights for technological innovation in other agricultural fields.

Although the proposed VD-CNN has achieved promising experimental results, several limitations and potential avenues for improvement remain. First, hyperspectral images are characterized by high dimensionality and strong inter-band correlations, posing challenges to model generalization. Enhancing the generalization capability through effective data augmentation strategies is therefore a valuable direction for future research. For instance, generating diverse and realistic training samples using techniques such as Generative Adversarial Networks (GANs) may further improve model robustness under complex real-world scenarios. Second, the computational complexity of VD-CNN remains relatively high. In practical applications, reducing inference time and improving computational efficiency are critical challenges that must be addressed. Future work could explore the integration of depthwise separable convolutions and lightweight network architectures to reduce computational overhead without sacrificing classification performance.

## Data Availability

The raw data supporting the conclusions of this article will be made available by the authors, without undue reservation.
